# A Randomized Trial of Comparing the Efficacy of Two Neurofeedback Protocols for Treatment of Clinical and Cognitive Symptoms of ADHD: Theta Suppression/Beta Enhancement and Theta Suppression/Alpha Enhancement

**DOI:** 10.1155/2017/3513281

**Published:** 2017-02-09

**Authors:** Arash Mohagheghi, Shahrokh Amiri, Nafiseh Moghaddasi Bonab, Gholamreza Chalabianloo, Seyed Gholamreza Noorazar, Seyed Mahmoud Tabatabaei, Sara Farhang

**Affiliations:** ^1^Research Center of Psychiatry and Behavioral Sciences, Tabriz University of Medical Sciences, Tabriz, Iran; ^2^Azarbaijan Shahid Madani University, Tabriz, Iran; ^3^Department of Physiology, Tabriz Branch, Islamic Azad University, Tabriz, Iran

## Abstract

*Introduction*. Neurofeedback (NF) is an adjuvant or alternative therapy for children with Attention Deficit Hyperactivity Disorder (ADHD). This study intended to compare the efficacy of two different NF protocols on clinical and cognitive symptoms of ADHD.* Materials and Methods*. In this clinical trial, sixty children with ADHD aged 7 to 10 years old were randomly grouped to receive two different NF treatments (theta suppression/beta enhancement protocol and theta suppression/alpha enhancement protocol). Clinical and cognitive assessments were conducted prior to and following the treatment and also after an eight-week follow-up.* Results*. Both protocols alleviated the symptoms of ADHD in general (*p* < 0.001), hyperactivity (*p* < 0.001), inattention (*p* < 0.001), and omission errors (*p* < 0.001); however, they did not affect the oppositional and impulsive scales nor commission errors. These effects were maintained after an eight-week intervention-free period. The only significant difference between the two NF protocols was that high-frequency alpha enhancement protocol performed better in suppressing omission errors (*p* < 0.001).* Conclusion*. The two NF protocols with theta suppression/beta enhancement and theta suppression/alpha enhancement have considerable and comparable effect on clinical symptoms of ADHD. Alpha enhancement protocol was more effective in suppressing omission errors.

## 1. Introduction

Attention Deficit Hyperactivity Disorder (ADHD) is a serious health issue and a proper treatment is necessary to treat or prevent behavioral, social, and academic problems [1, 2].

Different treatments which are employed to improve cognitive performance in children with ADHD have certain pros and cons [3]. Although pharmacological treatments are easily applicable and usually useful for treating ADHD, their long-term health effects are still under question [4, 5]. They are also associated with certain side effects such as sleep disturbance, loss of appetite, and growth suppression. Furthermore, psychosocial treatments including training parents and behavioral therapy are effective interventions but results are not maintained in long term [4, 6]. Regarding the limitations of available treatments, new treatment options are needed for ADHD.

Neurofeedback (NF) has been introduced to treat ADHD recently and is able to improve the attention level and alleviate the hyperactivity symptoms [7–12]. The process provides a mechanism by which the patient can normalize the cortical activity profile through decreasing slow wave activity and increasing fast wave activity. It is expected that compensation of the dysfunctional electroencephalogram (EEG) enhances concentration and attention and increases the arousal level [13–17]. In fact, patients will learn how to enhance the desirable EEG frequencies associated with relaxed attention and how to reduce the undesirable frequencies which are associated with under- or overarousal [18].

Despite therapeutic advantages of NF for patients with ADHD [10, 11, 19], the results of various studies are not conclusive. The reason for dissimilar results of available reports might be the different treatment techniques or protocols used for this purpose. In other words, since NF is almost a new treatment approach, a variety of treatment protocols is being examined. Different variations over different bands of cortical activity in various parts of the cortex have been applied, and each one has been accompanied by different clinical efficiencies. The most common treatment protocol for improving various symptoms of ADHD involves suppressing theta wave and enhancing beta wave [20]. Alpha activity might be an interesting target of treatment as it is associated with different types of cognitive processes, memory performance, perceptual performance, and intelligence. Klimesch et al. [21] and Escolano et al. [22] report effectiveness of upper alpha power in improving cognitive performance in ADHD. However results are not conclusive about the best protocol for ADHD. Increased alpha reference power is associated with large event-related desynchronization, better memory, and perceptual performance [23, 24]. High-frequency alpha band would improve the memory function in patients with ADHD [25] and previous reports suggest targeting theta and alpha activity in NF protocols [26].

According to previous findings, this study aims to compare the efficacy of two NF protocols including theta suppression/beta enhancement and theta suppression/high-frequency alpha enhancement regarding their effect on cognitive functioning of children with ADHD.

## 2. Methods and Materials

### 2.1. Participants

Sixty children with ADHD were randomly selected from patients who were referred to the specialized psychiatric clinics in Tabriz, northwest of Iran. This study was verified by the Scientific and Ethics Committee of Tabriz University of Medical Sciences as a doctoral thesis [27]. The protocol is registered in Iranian Registry of Clinical Trials (IRCT201404122660N4, http://en.search.irct.ir/view/17747). After a thorough explanation of the study purpose, caregivers of participants signed the consent form for participating in the study.

**Figure 1:**
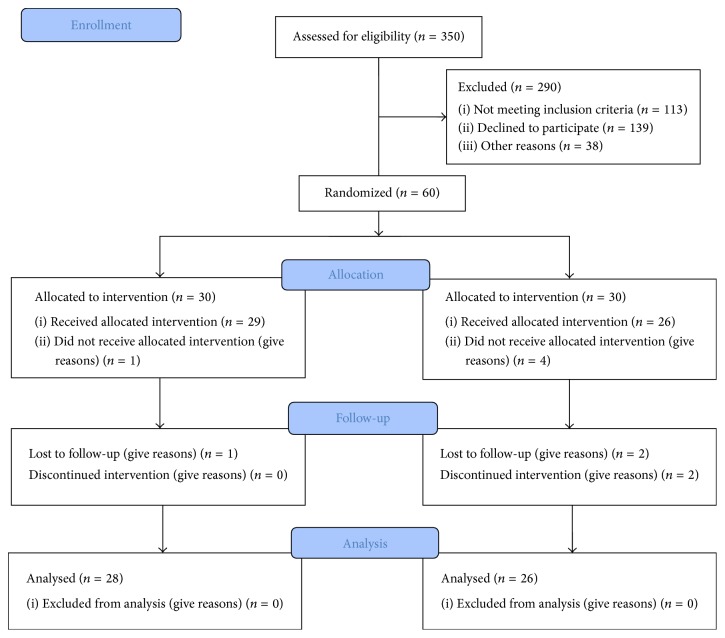
Flow diagram of the participants.

All of drug naïve children and adolescents who meet the DSM-5 diagnostic criteria (American Psychiatric Association, 2013) for combined ADHD were eligible. The diagnosis was made through a semistructured diagnostic interview with parents using SADS-K-PL by a child and adolescent psychiatrist. Exclusion criteria were history of severe head injury, neurological disorders, genetic disorders, psychiatric disorders other than ADHD, and intellectual disability. Children who had received psychotherapy within the past one year were also excluded. After enrolment by the evaluating psychiatrist, selected children were randomly divided into two groups by a schedule generated by RandList (Figure 1) by a nonevaluator coauthor. Recruitment started in April and ended in December 2014.

The NF protocol included theta suppression/beta enhancement in one group and theta suppression/high-frequency alpha enhancement in the other group. Children received forty sessions of NF (three sessions per week, 45 minutes each).

Clinical symptoms intensity and mental activity (by Quantitative Electroencephalography) were measured right before the intervention, after 40 sessions of NF, and after an 8-week intervention-free follow-up.

### 2.2. Measures

#### 2.2.1. Conners' Parent Rating Scale (CPRS)

CPRS was used to assess behavior of participants and the intensity of the symptoms of ADHD (Conners et al., 1998). Similarly, the computerized version of Conners' continuous performance task II (CPT-II) (Conners and Staff, 200) was used to analyze problems of inattention. Scores of omission and commission indicate the participants' performance in this test.

#### 2.2.2. Schedule for Affective Disorders and Schizophrenia for School Aged Children Present and Lifetime Version (K-SADS-PL)

This questionnaire is a semistructured diagnostic interview which has been designed based on DSM-IV criteria for current and past episodes of psychopathology. The interview includes children and their parents. Reliability and validity of the questionnaire have been confirmed in Iran [28].

#### 2.2.3. The ADHD Rating Scale

There are 18 symptoms of ADHD in this scale and its questions are answered based on 4 Point Likert Scale. Reliability and validity have been confirmed elsewhere [29]. This scale is sensitive to changes during treatment and is also suitable for research purposes.

#### 2.2.4. The Revised Conners' Parent Rating Scale (CPRS-R)

This test is used to assess core symptoms of ADHD and certain comorbidities such as oppositional disorder and conduct disorder. It is used for children and adolescents (3–17 years) and is able to separately assess inattention, hyperactivity, and impulsivity. This scale is used for both screening and monitoring the treatment results. Validity and reliability of the Persian version of CSR have been confirmed in Iran [30].

#### 2.2.5. Continuous Performance Test (CPT-II)

This test is very popular in assessing cognitive aspects of ADHD. Its main objective is analyzing the sustained attention as well as impulsivity. The Persian version of the test, which is computer based, has 150 Farsi digits which are considered as stimuli, out of which 30 stimuli (20%) are target stimuli and the remaining 80% are considered as nontarget stimuli. The interval between offering two stimuli is 500 ms and each stimulus is offered for 150 ms. The test period, considering the practical phase, which is carried out in order to enhance the subject's understanding before the main phase, is 200 s. Two types of errors are scored: omission and commission errors. The error of omission occurs when the subject fails to respond to the target stimulus indicating that the subject has had problem in understanding the stimulus, whereas the error of commission occurs when the subject responds to the nontarget stimulus. This reflects the deficiency in inhibiting the impulse. In this test, these two errors are counted by software. Correct responds and reaction time of the participant are also calculated. Reliability and validity of the Farsi version of CPT are reported in previous studies [31].

#### 2.2.6. Recording EEG and Neurofeedback Protocol

EEG was recorded with international 10-10 system in the same clinic where psychiatric evaluations were made. Data were recorded using Nihon Kohden Amplifier and with the sampling rate of 500 Hz, power-line notch-filtered at 50 z, and band-pass filtered at 0.5–60 Hz. EEG was recorded with open and closed eye, each for 10 minutes. Segmentation of EEG was considered as 2-second epochs. Artifacts caused by eyeball movements were refined using Gratton et al. algorithm (1983). Neuriguid software and Fourier transformation software were used to quantify EEG data. The relative power among all components resulting from quantitative analysis of the cortex activity was used.

Two protocols were applied for training NF: (1) enhancing high-frequency alpha protocol in frontocentral areas of brain and suppressing theta waves and (2) enhancing beta in frontocentral areas of brain and suppressing theta waves. The system was a Procomp Infinity from Thought Technology Ltd. (Montreal, QC, Canada) running Biograph Infinity software. The session included 5-minute baseline record and 20-minute intervention in frontal area (4 games, five minutes each) and 20 bipolar intervention therapies in central areas. In alpha protocol, participants needed to suppress theta waves (4–7 Hz) and to enhance the high-frequency alpha waves (10–12 Hz). In beta protocol, participants needed to suppress theta waves and to enhance low-frequency beta waves (12–15 Hz, SMR).

Feedback by the device was only provided when individual could distinguish the increase in high alpha or SMR.

#### 2.2.7. Statistical Analysis

Data were analyzed by SPSS (version 17). A General Linear Model (GLM) with repeated measurements was used to evaluate effects of the interventions on performance of participants. The type of intervention as between-subjects factor (group) and time of measurements as the within-subjects factor (time) were considered in clinical symptoms and cognitive performance. Descriptive information is presented as mean ± standard deviation and the level of significance was considered at 0.05.

## 3. Results

Each treatment group included 30 children and adolescents with ADHD. Six children failed to complete the course of treatment and were excluded from the analysis. The final sample consisted of 54 members with mean age (standard deviation) of 8.51 (1.44) years, including 28 children in alpha enhancement group (alpha group) and 26 children in beta enhancement group (beta group).

The two groups were matched in terms of gender, age, and disease duration. All of quantitative indices recorded by qEEG were also compared between the two groups prior to neurofeedback sessions and there was no significant difference.

No side adverse event was reported from participants or parents.

## 4. Clinical Symptoms

Table 1 indicates descriptive data on performance of participants of both groups in Conners' scale and CPT-II before and after intervention and 8 weeks after treatment completion. Analysis of Variance (ANOVA) with repeated measurements showed that both interventions had significant effect on scores of ADHD (*F* = 330.63, *p* < 0.001), hyperactivity (*F* = 198.49, *p* < 0.001), inattention (*F* = 491.36, *p* < 0.001), and omission (*F* = 15.30, *p* < 0.001), but effect was not significant on oppositional behavior. The group/time interaction was significant for omission errors (*F* = 19.39, *p* < 0.001) and alpha enhancement protocol made more suppression. These results were maintained after an 8-week follow-up.

## 5. Cortical Activity

Table 2 summarizes the descriptive data about variations of the relative power of various EEG bands activity in two anterior and posterior areas of brain through three modes: before intervention, after intervention, and eight weeks after treatment.

Groups showed different changes in delta band (*F* = 19.37, *p* < 0.001), high-frequency alpha band (*F* = 5.04, *p* = 0.05), and low-frequency beta band (*F* = 14.93, *p* < 0.001).

**Figure 2:**
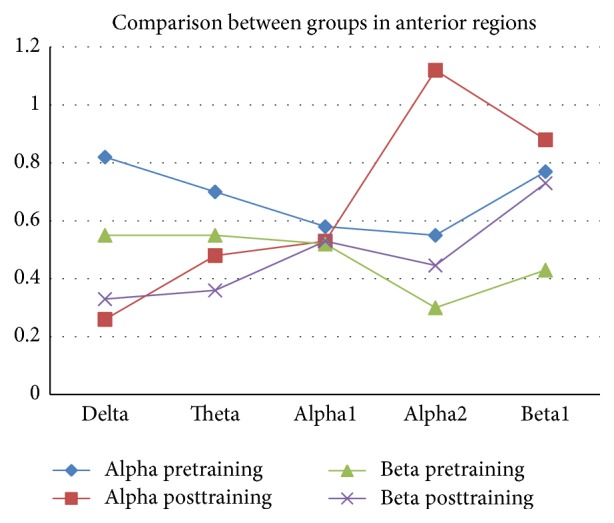
The cortical activity in anterior regions.

**Figure 3:**
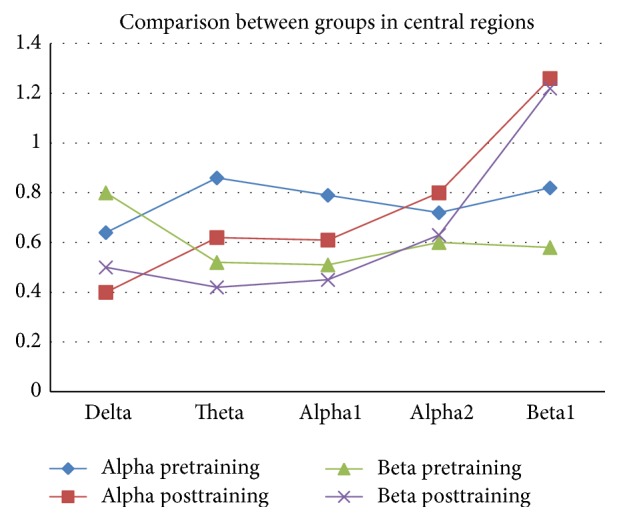
The cortical activity in central regions.

In regard to delta band, alpha group had more suppression in anterior areas. Likewise, in regard to high-frequency band, alpha group showed more enhancements in anterior areas compared to beta group. However, in regard to low-frequency beta band, beta group showed significant enhancement in posterior areas. Figures 2 and 3 depict cortical activity in anterior and central regions. In these two figures, powers of delta, theta, alpha1, alpha2, and beta1 bands are given in separate lines. The values before and after the training by the two protocols (alpha training and beta training) are also given in separate lines.

The results suggested that there was not a significant difference between the two groups in terms of intensity of theta (*F* = 1.03, NS) and low-frequency bands (*F* = 0.43, NS) in both anterior and posterior areas.

## 6. Discussion

As a very common disorder, evaluation of different approaches and choosing the best treatment are vitally important for ADHD [2]. NF is a treatment to improve the cognitive performance of patients with ADHD. As a nonpharmacological treatment with no side effect, NF has revived hopes of treating targeted disorders. However, since NF is still very young, several studies are still needed to come to a conclusion [13–16, 18]. Previous studies have suggested NF as a treatment option for children with ADHD whose parents prefer nonpharmacological treatments [10, 20].

A study showed that NF technique, through improving the sensorimotor rhythm (12–15 Hz) and beta activity (15–181 Hz), as well as medication (methylphenidate), has positive effect on improving inattention and speed and precision of continuous attention [10]. Previous studies also reported a significant improvement in ADHD symptoms after NF treatment in more than 3 out 4 children with ADHD [10, 32]. Most of researchers have evaluated beneficial effect of increasing beta (which reflects cognitive tasks) and decreasing theta which may coincide with ADHD and is related to hyperactivity, impulsivity, and inattentiveness. Alpha wave activity is related to cognitive activities of the brain [22, 33] and also there is a relation between better cognitive activity and higher alpha power during resting condition and the lower alpha power during cognitive tasks [34].

Based on high overlap of sensorimotor rhythm (12–15 Hz) with high-frequency alpha band activity as well as the strong correlation between high-frequency alpha band and cognitive performance improvement, Hanslmayr et al. supposed that the efficacy of NF on increasing sensorimotor rhythm protocol is due to the enhanced power of high-frequency alpha band [25]. They designed a protocol and observed that the enhanced power of high-frequency alpha and the suppressed theta band will result in improvement of memory performance in healthy people [25].

Another open label study reports that the enhancement of upper alpha power is effective in improving several measures of clinical outcome and cognitive performance in ADHD [22].

Results of our study are compatible with these reports and show efficacy of theta suppression/alpha enhancement on hyperactivity, inattention, and also omission errors. In the present study none of two protocols had an effect on oppositional and impulsive behaviors as well as the error of commission which reflect impulsivity. Leins et al. compared the NF treatment with theta/beta frequencies protocol and training slow cortical potentials and did not find any significant difference between the two considerably effective protocols in terms of behavioral or perceptual results [35]. However, in contrast to our results Gevensleben et al. observed that both slow cortical potential NF and theta/beta protocol have not positive effect on improving the behavioral problems related to disobedience and opposition [36]. Results of our study showed that both theta suppression/beta enhancement and theta suppression/alpha enhancement protocols were effective in reducing clinical symptoms. However high-frequency alpha enhancement protocol made more suppression in omission errors which reflect inattentiveness.

However, nonspecific factors may contribute to the positive effects induced by NF and improvement of the core symptoms of ADHD [37]. There are three nonspecific factors described in previous studies. These include the high amount of time spent with the therapist during NF, better motivation for changes in ADHD symptoms, and cognitive-behavioral training introduced under NF [34, 38]. These factors may explain some improvement of hyperactivity but may be a minor factor.

Absence of a control group might be the main limitation of the study. However, as stated by previous studies, ethical principles are against using a waiting or sham group for our treatment naïve patients. An active control group is suggested instead, but this will not solve the problem here, as the effect of confounding factors will not be distinguished. This is the reason for absence of a control group in several previous studies [35, 39, 40], as well as the present one. Another limitation of this study is that cortical activity during the session was not recorded. This issue is in fact similar to not having a control group because, without within-session data, it is still not clear whether the changes are a result of this specific intervention. However this situation is equal for both groups, and we have compared effect of the two protocols in a similar situation. Adding data obtained after a wash out period might also strengthen the relationship between the intervention and the result.

## 7. Conclusion

Both NF protocols were equally effective in alleviating the clinical symptoms of ADHD, as reported by parents. However, increasing high-frequency alpha protocol was associated with lower errors of omission.

## Figures and Tables

**Table 1:** Descriptive statistics of clinical symptoms and cognitive performance.

Scale	Group	Pretest	Posttest	Follow-up
ADHD	Alpha	42.28 ± 6.05	30.71 ± 7.43	31.14 ± 6.54
	Beta	43.80 ± 5.44	31.76 ± 5.46	31.84 ± 4.96
Hyperactivity	Alpha	13.85 ± 3.68	9.21 ± 3.31	9.71 ± 3.43
	Beta	14.65 ± 3.65	14.65 ± 2.05	9.42 ± 2.16
Inattention	Alpha	18.07 ± 3.55	9.07 ± 2.05	9.42 ± 2.16
	Beta	19.23 ± 2.73	11.53 ± 2.71	11.15 ± 3.04
Impulsivity	Alpha	9.14 ± 2.30	8.07 ± 1.92	7.85 ± 2.54
	Beta	9.11 ± 2.00	8.50 ± 1.97	7.19 ± 2.33
Omission	Alpha	9.25 ± 3.51	7.61 ± 3.47	7.95 ± 3.40
	Beta	7.72 ± 3.13	6.81 ± 2.79	7.21 ± 3.40
Commission	Alpha	21.51 ± 3.88	23.20 ± 4.14	22.76 ± 3.66
	Beta	20.69 ± 6.54	26.10 ± 2.63	27.02 ± 3.77

**Table 2:** Descriptive statistics on cortical activity in two groups.

Scale	Group	Brain area	Pretest	Posttest	Follow-up
Delta	Alpha	Frontal	0.82 ± 0.57	0.26 ± 0.20	0.29 ± 0.25
		Central	0.64 ± 0.38	0.40 ± 0.27	0.45 ± 0.31
	Beta	Frontal	0.55 ± 0.30	0.33 ± 0.30	0.31 ± 0.26
		Central	0.80 ± 0.48	0.50 ± 0.35	0.45 ± 0.31

Theta	Alpha	Frontal	0.70 ± 0.49	0.48 ± 0.39	0.45 ± 0.44
		Central	0.86 ± 0.58	0.62 ± 0.45	0.65 ± 0.49
	Beta	Frontal	0.55 ± 0.45	0.36 ± 0.26	0.38 ± 0.38
		Central	0.52 ± 0.36	0.42 ± 0.29	0.46 ± 0.32

Alpha 1	Alpha	Frontal	0.58 ± 0.29	0.53 ± 0.38	0.53 ± 0.40
		Central	0.79 ± 0.54	0.61 ± 0.48	0.64 ± 0.40
	Beta	Frontal	0.52 ± 0.36	0.53 ± 0.45	0.57 ± 0.44
		Central	0.51 ± 0.41	0.45 ± 0.30	0.47 ± 0.494

Alpha 2	Alpha	Frontal	0.55 ± 0.38	1.12 ± 0.30	0.89 ± 0.44
		Central	0.72 ± 0.40	0.80 ± 0.41	0.77 ± 0.47
	Beta	Frontal	0.30 ± 0.35	0.46 ± 0.39	0.42 ± 0.37
		Central	0.60 ± 0.57	0.63 ± 0.51	0.67 ± 0.49

Beta 1	Alpha	Frontal	0.77 ± 0.55	0.88 ± 0.57	0.86 ± 0.49
		Central	0.82 ± 0.60	1.26 ± 0.46	0.878 ± 0.75
	Beta	Frontal	0.43 ± 0.31	0.73 ± 0.70	0.69 ± 0.64
		Central	0.58 ± 0.56	1.22 ± 0.67	0.84 ± 0.44
